# Using text analysis to quantify the similarity and evolution of scientific disciplines

**DOI:** 10.1098/rsos.171545

**Published:** 2018-01-17

**Authors:** Laércio Dias, Martin Gerlach, Joachim Scharloth, Eduardo G. Altmann

**Affiliations:** 1 Max Planck Institute for the Physics of Complex Systems, 01187 Dresden, Germany; 2 Department of Chemical and Biological Engineering, Northwestern University, Evanston, IL 60208, USA; 3 Department of German, TU Dresden, Applied Linguistics, 01062 Dresden, Germany; 4 School of Mathematics and Statistics, University of Sydney, Sydney 2006, New South Wales, Australia

**Keywords:** science of science, information theory, dissimilarity measures

## Abstract

We use an information-theoretic measure of linguistic similarity to investigate the organization and evolution of scientific fields. An analysis of almost 20 M papers from the past three decades reveals that the linguistic similarity is related but different from experts and citation-based classifications, leading to an improved view on the organization of science. A temporal analysis of the similarity of fields shows that some fields (e.g. computer science) are becoming increasingly central, but that on average the similarity between pairs of disciplines has not changed in the last decades. This suggests that tendencies of convergence (e.g. multi-disciplinarity) and divergence (e.g. specialization) of disciplines are in balance.

## 1. Introduction

The digitization of scientific production opens new possibilities for quantitative studies on scientometrics and science of science [1], bringing new insights into questions such as how knowledge is organized (maps of science) [2–6], how impact evolves over time (bibliometrics) [7,8], or how to measure the degree of interdisciplinarity [9,10]. At the heart of these questions lies the problems of identifying scientific fields and how they relate to each other. The difficulty of these problems, and the inadequacy of a purely essentialist approach, were clear already to Popper [11]: ‘The belief that there is such a thing as physics, or biology, or archaeology, and that these “studies” or “disciplines” are distinguishable by the subject matter which they investigate, appears to me to be a residue from the time when one believed that a theory had to proceed from a definition of its own subject matter. But subject matter, or kinds of things, do not, I hold, constitute a basis for distinguishing disciplines.’ [11], pp. 124–125. Instead, he argued that disciplines have a cognitive and a social dimension [12], i.e. they ‘are distinguished partly for historical reasons and reasons of administrative convenience (such as the organization of teaching and of appointments), and partly because the theories which we construct to solve our problems have a tendency to grow into unified systems.’ [11], p. 125.

On the one hand, the social dimension of scientific fields can be defined in terms of different institutions establishing stable recurring patterns of behaviour [13]: producing and reproducing institutions such as research institutes and universities, communicative institutions such as scientific societies, journals or conferences, collecting institutions (journals, libraries), as well as directing institutions (ministries, scientific advisory boards), etc. All these institutions contribute to the formation, stabilization and reproduction of a discipline as well as its distinction from others. On the other hand, the cognitive dimension has been specified in [13] as a number of fundamental invariants in the procedural knowledge, which lead to the categorical construction of scientific knowledge. If this process causes a change in the cognitive realm for an object of knowledge, it constitutes a certain discipline.

The brief discussion above is sufficient to show that both the definition and relation between scientific fields depend on multiple dimensions (e.g. essentialist, social and cognitive). There is no correct or intrinsically better classification or organization of scientific fields. The consequence of this view to the problem of identifying relationships between scientific fields from empirical data is that there is no *ground truth* against which results (e.g. clusters) can be compared [14]. While previous quantitative analyses often aimed at obtaining measures which lead to *better* classification methods [15], the approach we pursue here is to design methods with a clear interpretation and with known statistical properties in order to quantify and understand the different aspects captured by different dimensions. This approach is in line with very recent work in scientometrics [6].

Traditional (expert) classifications are mostly motivated by the ‘*subject matters*’ under investigation and can be associated with an essentialist view. The empirical analysis of citation networks, an approach with a long tradition in scientometrics [16,17], can be regarded as capturing the social dimension (i.e. collecting institutions in the form of journals). While citations offer valuable insights into the structure and dynamics of science, they thus reflect only one particular dimension of the relationship between publications (or scientists) largely ignoring the actual content of the scientific articles. By contrast, the cognitive dimension can be operationalized with the help of linguistic features (e.g. keywords as indicators for conceptual imprints of disciplines). The increasing availability of full text of scientific articles (e.g. of Open Access journals) provides new opportunities to study the latter aspect in the form of written language. Examples include: (i) the tracking of the spread of ideas [18], individual words (memes) [19], or scientific concepts [20]; (ii) quantifying differences in the scientific discourse between subdomains in biomedical literature [21] or ‘hard’ and ‘soft’ science [22]; and (iii) efforts to combine citation and textual information [2,23–26].

In this work, we advance the idea that the organization and evolution of science should be studied through different, complementary dimensions. We add a new methodology that provides a meaningful, language-based, organization of scientific disciplines based on written text. We perform a systematic analysis how this compares with the organization obtained from experts and citations and, furthermore, trace the temporal evolution in the relation between different scientific disciplines.

More specifically, we introduce an unsupervised methodology to analyse the text of scientific articles. Our methodology is based on an information-theoretic dissimilarity measure we proposed recently [27] (more technically, it is a generalized and normalized Jensen–Shannon divergence between two corpora). The main advantage of this measure is that it has an absolute meaning (i.e. it is not based on relative comparisons) and it is statistically more robust than traditional approaches [27,28], e.g. with respect to the detection of spurious trends owing to rare words and increasing corpus sizes. We measure the similarity between scientific fields based on ≈10^7^ abstracts from the last three decades (Web of Science database). Comparing our language analysis to a citation analysis and an experts classification, we find that the language and citation are more similar to each other but the language is even more distinct from the experts than the citation analysis. Following the relation between scientific fields over time, our language analysis reveals the scientific fields that are becoming more central in science. However, overall (averaged over all pairs of disciplines) we find that the similarity between the language of different fields is not increasing.

## 2. Dissimilarity of scientific fields

We are interested in the general problem [2,4] of quantifying the relationship between two scientific fields *i*,*j* through the computation of a dissimilarity measure *D*(*i*,*j*), i.e. a quantification of how different *i* and *j* are. Dissimilarity measures are symmetric *D*(*i*,*j*)=*D*(*j*,*i*), non-negative *D*(*i*,*j*)≥0 and *D*(*i*,*i*)=0 [29]. Each scientific field is defined by (at least hundreds of) papers classified by Web of Science as belonging to the same category (see Material and methods §5.1 for details on the data). We consider dissimilarities computed from the following three different types of information (expert, citation and language).

### 2.1. Experts

The classification of disciplines by their relationship is as old as science itself. The most used structure is a strict hierarchical tree, as seen in the traditional departmental division of universities. The collection of papers used here, provided by ISI Web of Science [30], provides a classification of papers according to the Organisation for Economic Cooperation and Development (OECD) classification of fields of science and technology [31]. This scheme is a hierarchical tree with scientific fields defined at three levels (domains, disciplines and specialities). For instance, *Applied Mathematics* (a speciality) is part of *Mathematics* (a discipline) which is part of *Natural Sciences* (a domain). The natural dissimilarity measure *D*_exp_(*i*,*j*) between two fields in this structure is the number of links needed to reach a common ancestor of *i* and *j*. For instance, considering *i*,*j* at the specialty level, *D*_exp_ can assume three different values: *D*_exp_=1 for specialties belonging to the same discipline (e.g. *Applied Mathematics* and *Statistics & Probability*), *D*_exp_=2 for specialties belonging to the same domain (e.g. *Applied Mathematics* and *Condensed Matter Physics*) and *D*_exp_=3 for the other pairs of specialties (e.g. *Applied Mathematics* and *Linguistics*). While researchers have pointed out potential issues with classification into categories of ISI Web of Science [4], it offers the most extensively available classification and remains widely used to relate articles and journals to disciplines [9,32].

### 2.2. Citations

Another popular approach is to consider that fields *i* and *j* are more similar if there are citations from (to) papers in *i* to (from) papers in *j* [4,16,17]. Here, we consider a dissimilarity measure *D*_cite_(*i*,*j*) which decreases for every citation between papers in *i* and *j* and increases with every citation from *i* that is not to *j* (and vice versa), but that remains unchanged by the number of citations that do not involve either *i* or *j*. These requirements are achieved using (for *i*≠*j*) a symmetrized Jaccard-like dissimilarity [29,33]:

2.1 $$D_{\mathrm{cite}}(i,j)=\frac{1}{2}\left(\frac{C_{i,\bar{j}}+C_{\bar{i},j}}{c_{i,j}+C_{i,\bar{j}}+C_{\bar{i},j}}+\frac{C_{j,\bar{i}}+C_{\bar{j},i}}{c_{j,i}+C_{j,\bar{i}}+C_{\bar{j},i}}\right),$$

where *c*_*i*,*j*_ are the number of citations from *i* to *j*, $C_{a,\bar{b}}=\sum_{t=1,t\neq b}^{N}c_{a,t}$ and $C_{\bar{a},b}=\sum_{t=1,t\neq a}^{N}c_{t,b}$^1^.

### 2.3. Language

We compare the language of fields *i* and *j* based on the frequency of words in each field using methods from information theory. Measuring the frequency *p*(*w*) of word *w*, for each field *i* we obtain a vector of frequencies **p**_*i*_≡*p*_*i*_(*w*) for *w*=1,…,*V* , where *V* is the size of the vocabulary (i.e. number of different words). From this, following [27], the dissimilarity between two fields *i* and *j* is

2.2 $$D_{\mathrm{lang}}(i,j)=\frac{2H_{2}({\text{p}}_{i}+{\text{p}}_{j})/2-H_{2}({\text{p}}_{i})-H_{2}({\text{p}}_{j})}{\left(1/2)(2-H_{2}({\text{p}}_{i})-H_{2}({\text{p}}_{j})\right)},$$

where $H_{2}({\text{p}}_{i})=1-\sum_{w}p_{i}{\left(w\right)}^{2}$ is the generalized entropy of order 2 and the denominator ensures normalization (i.e. 0≤*D*_lang_(*i*,*j*)≤1). To increase the discrimination power and to avoid statistical biases in our estimation, we removed a list of stop words and included only the *V* =20 000 most frequent words (see Material and methods §5.3 for a justification). The dissimilarity (2.2) corresponds to a generalized (and normalized) Jensen–Shannon divergence which yields statistically robust estimations in texts [27,28] (for details and motivation, see Material and methods §5.4).

In contrast with most previously proposed methods, equation (2.2) has two critical properties that are essential in order to obtain the interpretable results mentioned in the Introduction. On the one hand, it is well founded in information theory and its statistical properties (in terms of systematic and statistical errors) are well understood [27,34], distinguishing it from other heuristic approaches. On the other hand, it has convenient properties: *D*_lang_(*i*,*j*) depends only on the papers contained in fields *i* and *j* and it is normalized 0≤*D*_lang_(*i*,*j*)≤1. As a result, the measured distance between two fields, *D*_lang_(*i*,*j*), has an absolute meaning. This is in contrast with alternative similarity measures [2,4], including machine-learning approaches (e.g. topic models [15,35]) based on (un-) supervised classification of documents into coherent subgroups. Here, the main limitations stem from the fact that either (i) the division into subgroups is typically based on statistically significant differences in the usage of words between the different subgroups independent of the actual effect size, or (ii) the resulting distance between two fields depends also on all other fields (e.g. the distance between ‘*Physics*’ and ‘*Chemistry*’ depends on whether one includes articles about ‘*Anthropology*’ in the classification).

## 3. Results

We now present and interpret results obtained computing the three dissimilarity measures (*D*_exp_,*D*_cite_ and *D*_lang_) reported above for scientific fields *i*,*j* defined by papers published in different time intervals and categorized (by Web of Science) as belonging to the same specialty (e.g. *Applied Mathematics*), discipline, (e.g. *Mathematics*) or domain (e.g. *Natural Sciences*).

### 3.1. Comparison of dissimilarity measures

**Figure 1.**
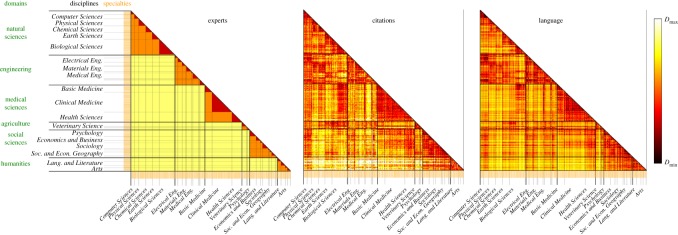
Dissimilarity between specialties measured in three different dimensions: (*a*) *D*_exp_ based on experts classification [31], where $D_{\min}=0$ and $D_{\max}=4$; (*b*) citations dissimilarity *D*_cite_ (2.1), where $D_{\min}=0$ and $D_{\max}=7.5$; (*c*) language dissimilarity *D*_lang_ (2.2), where $D_{\min}=0$ and $D_{\max}=1$. *N*=225 specialties of the OECD classification scheme are considered. Results based on ≈21 M papers from 1991 to 2014 (see §5.1 for details).

Figure 1 shows the three *D*(*i*,*j*) at the level of specialties (*i*,*j*) for the complete time interval 1991–2014. The concentration of low *D*(*i*,*j*) close to the diagonal shows that both the citations and language of scientific papers partially reflect the disciplinary classification done by the experts. However, visual inspection already reveals that citations and our language analysis show relationships not present in the expert classification, e.g. the low dissimilarity between *Engineering* and *Natural Sciences* (most clearly between *Electrical Engineering* and *Physical Sciences*) and between the disciplines inside the *Agriculture* domain and *Biological Sciences*.

We start by quantifying the relationship between the three different dissimilarity measures, i.e. (*D*_exp_,*D*_cite_ and *D*_lang_), across all pairs of specialties (*i*,*j*). In table 1, we report the rank correlation between the three measures, which we obtain from ranking for each dissimilarity the pairs of (*i*,*j*) according to *D*(*i*,*j*). The choice of this non-parametric correlation is motivated by the fact that the range of the three measures differs dramatically (e.g. *D*_exp_∈{0,1,2,3} and *D*_lang_∈[0,1]). The positive statistically significant correlation between all pairs of *D*(*i*,*j*)’s confirms the visual impression described above. The correlation between citations and language is higher than the correlation with the experts classification. Remarkably, language and citations show a very similar correlation with experts, but language is systematically less correlated than citations (*p*-value=1.8×10^−5^ for Spearman-*ρ* and *p*-value=2.2×10^−5^ for Kendall-*τ*^2^). We conclude that the language dissimilarity *D*_lang_ introduced here is able to retrieve the well-known relationships between disciplines to a similar extent with that of the (well-studied) citation analysis.

**Table 1.** Rank correlation between the dissimilarities *D*_*x*_(*i*,*j*) obtained from different dimensions *x*∈{exp (experts),cite (citations),lang (language)} computed over all specialty pairs (*i*,*j*). (All values are significantly different from zero (*p*-values <10^−5^). The two values in each cell denote the Kendall-*τ* and Spearman-*ρ* (in parenthesis). Qualitatively equivalent results are obtained in three different time intervals (indicated in the left row).)

time	lang-cite	lang-exp	cite-exp
all, 1991–2014	0.57 (0.76)	0.32 (0.39)	0.34 (0.42)
first half, 1991–2002	0.60 (0.80)	0.34 (0.41)	0.37 (0.46)
second half, 2003–2014	0.64 (0.84)	0.35 (0.43)	0.38 (0.47)

**Figure 2.**
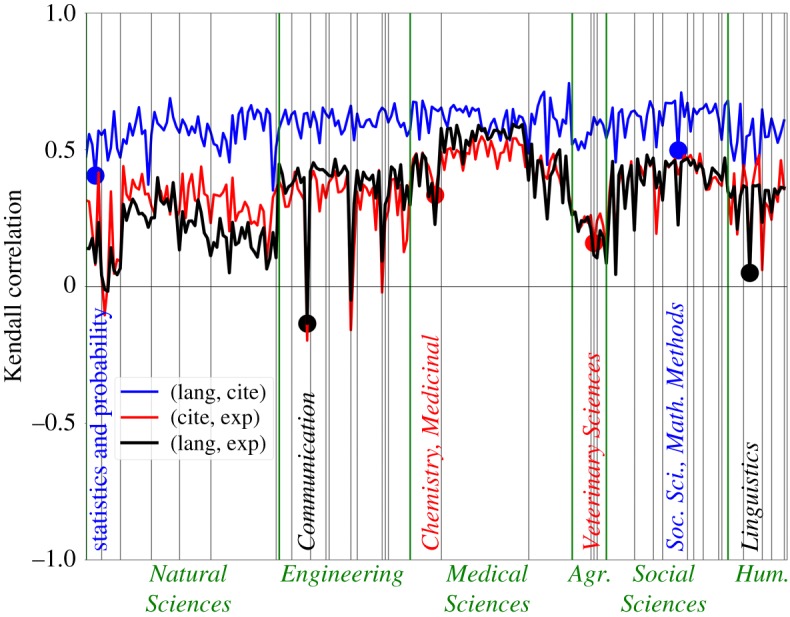
Correlation between the different dissimilarity measures varies across fields. The Kendall correlation *τ*(*x*,*y*) (shown in the vertical axis) for two measures *x* and *y* is computed between *D*_*x*_(*i*,*j*) and *D*_*y*_(*i*,*j*) over all specialties *j* for a fixed specialty *i* (shown in the horizontal axis). The three possible comparisons (*x*,*y*) are indicated in the caption. Six specialties (one from each domain) with low correlation are highlighted.

We now explore how the relationship between the different dimensions depends on the different scientific fields. The results in figure 2 confirm the conclusions of the aggregated analysis but show further interesting features. First, the correlation in (*D*_exp_,*D*_lang_) is smaller than (*D*_exp_,*D*_cite_) mainly in the natural sciences. Second, while the correlation between citations and language remains largely constant, large fluctuations in the correlations between expert and citations (as well as expert and language) exist. This is seen both as the strong downward spikes and also in the manifested dependence on disciplines and domains. The titles of the specialties at the low peaks already suggest that these are specialties with interdisciplinary connections. For instance, *Chemistry, Medicinal* is a specialty that (according to the experts classification) belongs to the discipline *Basic Medicine* and to the domain *Medical Science*. Therefore, *D*_exp_=3 between *Chemistry, Medicinal* and all specialties of the *Natural Sciences* (in particular, for all specialties from the discipline *Chemical Sciences*). Instead, the dissimilarity measured by citations *D*_cite_ and language *D*_lang_ yields much smaller values revealing the proximity of *Chemistry, Medicinal* to the *Natural Sciences,* thus explaining the low correlation in (*D*_exp_,*D*_cite_) and in (*D*_exp_,*D*_lang_). The other downward spikes seen in the figure can also be understood as a consequence of the central role played by the *Natural Sciences* in other disciplines. To confirm this, we computed for a list of selected specialties *i*=*i*^*spikes*^ the pairs (*i*,*j*) which suffered the largest change in ranks (comparing *D*_exp_ with *D*_lang_), finding that nine from the top 10 specialties were from the domain *Natural Sciences* (five of them from the discipline *Chemical sciences*, including the top two specialties).

### 3.2. Hierarchical clustering

**Figure 3.**
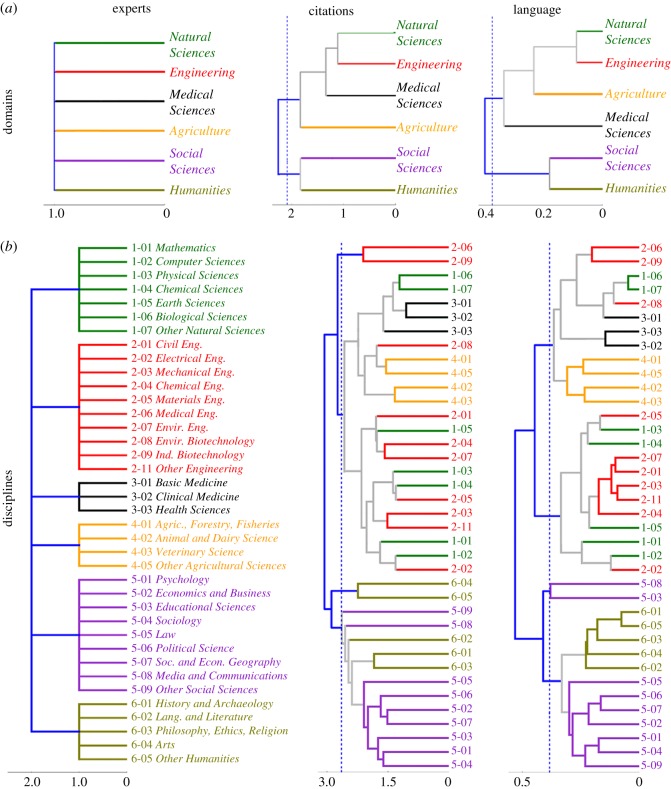
Hierarchical clusterings at the level of domains (*a*) and disciplines (*b*). Results for citations (language) were obtained by agglomerative hierarchical clustering, applying the group average method [36] to *D*_cite_(*i*,*j*) (*D*_lang_(*i*,*j*)). The *x*-axis shows the clustering dissimilarity (i.e. the dissimilarity of two clusterings that are merged). The dashed line corresponds to a clustering dissimilarity equal to the percentile 0.92 of the values of all cluster dissimilarities at each measure (citations/language).

A strict hierarchical classification of scientific fields is both aesthetically appealing and of practical use in bibliographical and document classification tasks. It also allows us to further highlight the differences in the relationship between scientific fields revealed by the different dissimilarity measures (in particular by *D*_lang_). While *D*_exp_ is precisely based on one such hierarchical classification, *D*_cite_ and *D*_lang_ are not. In figure 3, we show the hierarchical classifications induced by *D*_cite_ and *D*_lang_ through the computation of a simple clustering method at the level of domains and disciplines.

At the top level of the six domains (figure 3*a*), the clustering obtained from citations and from language are very similar. In particular, both identify *Engineering*-*Natural Sciences* and *Humanities*-*Social Science* as clusters that separate from the other domains in a similar fashion. The only difference is that, based on citations, *Agriculture* appears more isolated, while based on language this happens for *Medical Science*. A more detailed picture of the differences between language and citation is revealed at the level of disciplines (figure 3*b*). While at the first division, both citations and language create a cluster in which all disciplines of the domains *Humanities* and *Social Sciences* appear, further divisions show more subtle differences between the two dissimilarity measures.

Remarkably, the hierarchy obtained from language creates a cluster containing all and only *Humanities* disciplines. By contrast, the hierarchy based on citations creates one clustering with three of the five *Humanities* disciplines (*Lang. and Literature*, *Arts* and *Other Humanities* while the two remaining ones (*History & Archaeology* and *Philosophy, ethics, religion*) are clustered together in the middle of a cluster of disciplines in *Social Science*. Another interesting difference between the clusterings is revealed looking at three disciplines of the domain *Medicine*: In the analysis based on citations, the minimum cluster that includes the three disciplines includes *Biological Sciences* and *Other Natural Sciences*, while in the language analysis, this cluster includes additionally three related *Engineering* disciplines (*Medical Eng.*, *Ind. Biotechnology* and *Environ. Biotechnology*).

Probably the most remarkable feature of the clustering obtained by, both, citations and language is that it repeatedly clusters together related disciplines from *Natural Sciences* with disciplines from *Engineering* and *Medicine* (e.g. *Chemical Sciences* and *Materials Science*). This clustering, not present in the experts classification, suggests that the distinction between fundamental and applied sciences present in the expert classification has no strong effect on citations and the language of the publications. Instead, in this specific case, the citation and language analysis seem to be capturing a connection between ‘subject matters’ that was necessarily absent from the strict hierarchical expert classification.

### 3.3. Temporal evolution

**Figure 4.**
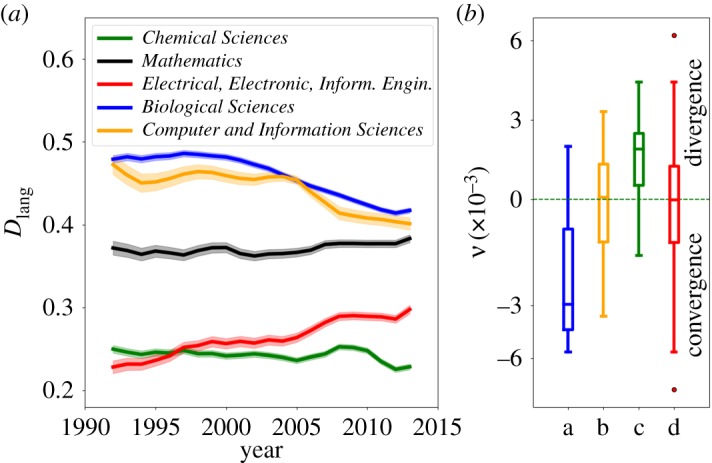
Evolution of the similarity between disciplines in the last three decades. (*a*) Distance *D*_lang_(*i*,*j*) between *Physical Sciences* (*i*) and other five selected disciplines (*j*) (3-year moving averages). (*b*) Total variation *ν*—defined in equation (3.1)—of the distance for pairs of disciplines with histories longer than 12 years. Each boxplot corresponds to the distribution of *ν* for pairs of disciplines where we fixed one of the disciplines. At position (*a*) we fixed *Computer and Information Sciences*, at (*b*) *Chemical sciences*, at (*c*) *Psychology* and at (*d*) we used all pairs of disciplines.

While in the previous sections we looked at a static snapshot of the relation between disciplines, here we are interested in how the linguistic relationship *D*_lang_(*i*,*j*) between pairs (*i*,*j*) of disciplines evolved over the last three decades.^3^ In figure 4, we show the temporal evolution for five out of 703 pairs (*i*,*j*), with focus on the discipline *Physical Sciences*, illustrating different types of dynamic patterns. On the one hand, the dissimilarity to *Chemical Sciences* (its most similar discipline) and *Mathematics* stays roughly constant over time. On the other hand, we also observe systematic trends of disciplines becoming more or less similar over time. While the proximity to *Biological Sciences* and *Computer and Information Science* has steadily increased (decreased dissimilarity *D*_lang_(*i*,*j*)) after the year 2000, the opposite trend is seen for *Electrical, Electronical and Information Engineering*. These observations are consistent with the increasing number of biological and computational-related publications in *Physics*, and with a departure from the historical connections to *Engineering*.

The observations reported above raise the question as to whether scientific disciplines are showing an overall tendency to become more similar to each other. In a more general context, this amounts to the question of whether the purported increase in *interdisciplinarity* leads to a larger overlap in the language used by different disciplines. We address this question by computing, for each pair of disciplines, the mean yearly variation

3.1 $$\begin{array}{r} \nu (i,j)=\frac{1}{\Delta t}\sum_{t\in \Delta t}D_{\mathrm{lang}}^{\left(t\right)}(i,j)-D_{\mathrm{lang}}^{\left(t-1\right)}(i,j) \end{array}$$

3.2 $$\begin{array}{r} =\frac{1}{\Delta t}(D_{\mathrm{lang}}^{\left(t_{f}\right)}(i,j)-D_{\mathrm{lang}}^{\left(t_{0}\right)}(i,j)), \end{array}$$

where the time interval Δ*t*≡*t*_*f*_−*t*_0_ was usually from *t*_0_=1991 to *t*_*f*_=2014. The distribution of values of *ν* for all discipline pairs (*i*,*j*) is shown at the (rightmost) box plot in figure 4*b*. We see that there are both positive and negative variations, consistent with our qualitative observations in the example of *Physical Sciences* in figure 4*a*. However, the average variation 〈*ν*〉≈−0.00025 over all pairs of disciplines (*i*,*j*) is not distinguishable from zero (the null hypothesis of 〈*ν*〉=0 has a *p*-value=0.07 in the *t*-test for the mean of one sample and a *p*-value=0.21 in the non-parametric Wilcoxon test), i.e. the typical dissimilarity remains unchanged. This result suggests that, while there are systematic trends for individual pairs of disciplines, on average there is no significant increase or decrease in the interdisciplinarity for the science as a whole in the last three decades as measured by the language.

On a more fine-grained level, however, we observe systematic trends that suggest that individual disciplines tend to become more (less) central. For this, we focus on the discipline pairs (*i*,*j*) which experienced the most extreme variation in the last decade (one standard deviation away from 〈*ν*〉). These pairs have typically |ν|⪆0.003, meaning that their (normalized) dissimilarity changes roughly 3% in a decade. The three disciplines that are most frequently seen in the left tail (*ν*<0) are: *1-02 Computer and Information Sciences*, *2-08 Environmental Biotechnology* and 3-01 *Basic Medicine*. The language of these disciplines became significantly more similar to the language of other disciplines in the last three decades, suggesting that these disciplines became more central. By contrast, the three disciplines that experienced most strongly the opposite effect (most frequently seen in the right tail, *ν*>0) are (in decreasing order): 5-01 *Psychology*, 2-05 *Materials Engineering* and 2-02 *Electrical Engineering, Electronic Engineering, Information Engineering*.

In the interpretation of the results reported in this section it is crucial to take into account that the measure *D*_lang_ we use depends only on the frequency of the words in each of the fields and in each year. In particular, this means that the results can be interpreted as an absolute dissimilarity independent of the content or volume of other fields. Another advantage of our measure *D*_lang_ is that it allows us to quantify the contribution of individual words [28]. This general feature of our method is illustrated in table 2 and allows for a deeper interpretation of the meaning of *D*_lang_ (e.g. the contribution of topical words and stylistic differences).

**Table 2.** Contribution of individual words *w* to the dissimilarity between *Physical Sciences* (*p*) and *Computer and Information Sciences* (*c*) in years 1991 (left) and 2014 (right). (The 10 words *w* that contributed most to *D*_lang_ in each year are shown. The relative contribution *C* is measured as the fraction of the total *D*_lang_ owing to word *w*. It is computed dividing the absolute contribution of word *w* to *D*_lang_—obtained from equation (2.2) by using the single *w* in the numerator and all *w*’s in the denominator (the normalization factor)—by the total *D*_lang_ leading to *C*=0.5(*p*_c_(*w*)−*p*_p_(*w*))^2^/(2*H*_2_((**p**_p_+**p**_c_)/2)−*H*_2_(**p**_p_)−*H*_2_(**p**_c_)). The last column shows the ratio of frequencies of word *w* in the two disciplines.)

year=1991, *D*^(1991)^_lang_(*p*,*c*)=0.49			year=2014, *D*^(2014)^_lang_(*p*,*c*)=0.40		
word *w*	*C*	*p*_c_(*w*)/*p*_p_(*w*)	word *w*	*C*	*p*_c_(*w*)/*p*_p_(*w*)
system	9.9%	3.981	algorithm	6.4%	14.134
algorithm	6.7%	30.196	propose	5.9%	5.091
problem	4.4%	10.355	problem	3.5%	11.388
paper	3.5%	7.782	paper	3.3%	5.107
temperature	2.3%	0.015	method	2.6%	2.246
language	2.2%	488.726	approach	2.6%	4.377
program	2.0%	34.313	data	2.5%	2.934
energy	1.9%	0.025	network	2.3%	11.313
field	1.5%	0.141	system	2.0%	2.079
set	1.3%	6.738	temperature	1.6%	0.015

## 4. Discussion

We investigated the similarity between scientific fields from different perspectives: an expert classification, a citation analysis and a newly proposed measure of linguistic similarity. We found that these different dimensions are related yet different, yielding thus new insights on the relationship between disciplines, their hierarchical organization and their temporal evolution.

Our first main finding is that the language and citation relationships between disciplines are similar and substantially different from the expert classification. This is consistent with the motivation exposed in our introduction which associated the expert classification to the (largely idealized) essentialist view of scientific disciplines, while the citation (social) and language (cognitive) were closer to dimensions that play a more important role in the relationship between fields. Interestingly, our results indicate that the language-relation of fields is more distinct from the expert classification than the citation-relation is, especially in the natural sciences.

Our second main finding is that in the last 30 years the language of different scientific fields remain, on average, at the same distance from all other fields. While individual disciplines show clear trends of increasing (or decreasing) centrality, this suggests that, overall, diverging tendencies in science (e.g. specialization) are in balance with converging tendencies (e.g. multidisciplinarism). This is a remarkable quantitative finding because of the substantial changes observed in this period.

The latter result demonstrates that our textual measure is of practical relevance for the study of interdisciplinarity. In recent years, interdisciplinary research achieved a central position [10] due to its broader relation to the concept of diversity [37] and its effect on the impact [38,39] and performance of teams [40] as well as its implications for policymaking, e.g. in terms of funding [41]. Is it just a fashion or science is really getting more and more interdisciplinary? A usual way to assess interdisciplinarity is based on citation networks using heuristic approaches [9,32,42] or methods from complex networks [43–46]. In line with the arguments exposed in the introduction, interdisciplinarity can be viewed through different dimensions and the cognitive dimension would be best measured using textual data. However, there are only very few works [47–49] relating textual measures with interdisciplinarity, despite the increasing availability of the text of scientific articles. In this view, the significance of our approach is that it provides a measure of interdisciplinarity based on how much the usage of words in different disciplines overlap.

Finally, we hope our results and methodology will stimulate a multiple-dimensional approach in other problems related to the study of sciences, profiting from the modern availability of large (textual) databases of scientific publications that allow us to go beyond traditional bibliometric analysis [1,9]. These include, but are not limited to, the formulation of more meaningful bibliometric indicators [50], the identification and prediction of influential papers and disciplines [51–53], or the inclusion of textual information in recommending related scientific papers [54].

## 5. Material and methods

### 5.1. Data and grouping of corpora

We use the Web of Science database [30] and explore the following information available for individual articles: citations, title, abstract and the classification in one scientific speciality (per OECD classification [31]). We use all papers published between 1991 and 2014 because, the number of articles with text in the abstract is substantial only after 1991 and because, at the time we started our analysis, 2014 was the last complete year available to us. The text of an article was built concatenating its title and abstract. The corpus representing a speciality in a given year is obtained from the concatenation of the text of all articles for that specialty in that year. The corpus for one discipline (or domain) concatenates all articles in all specialities belonging to that discipline (or domain).

Our analysis is based on 19 589 166 articles for each of the textual and classification information were available (92% of all articles indexed in Web of Science during 1991–2014). In our analysis we considered only citations from and to the papers in our list because only for these papers we had a reliable classification of specialties. These citations corresponded to roughly half of the ≈625 M citations associated with these papers. See [55] for the divergences we obtained from this dataset.

### 5.2. Data processing

For each article in our database, we performed the following steps to process the textual information:

(i) the abstracts written in a language different from English were excluded;
(ii) the copyright information contained in the abstract was removed;
(iii) title and abstract were concatenated;
(iv) the text was converted to lowercase;
(v) contractions were replaced by their non-contracted form, exact list and details are available in [55];
(vi) the text was tokenized, and the nouns and verbs were lemmatized using the Natural Language Toolkit [56] for English;
(vii) non-alphanumeric symbols (except hyphen) inside tokens were replaced by white space, therefore generating two or more distinct tokens;
(viii) tokens composed exclusively by numbers or single letter were removed; and
(ix) tokens belonging to a preset stop-word list were discarded; exact list and details available in [55].

### 5.3. Minimum corpus size

We computed *D*_lang_ using only the 20 000 most frequent word types, disregarding the scientific fields for which there were not enough data to achieve this cut-off. This is necessary in order to ensure that the estimations of *D*_lang_ are reliable. The crucial problem is the slow convergence of entropy estimations (and thus *D*_lang_) which leads to strong uncertainties in entropy estimations even for large corpora [27]. By choosing a fixed number of word types, we reduce the effect of the remaining bias (in the estimation of *D*_lang_) on our comparative analysis of textual dissimilarity between pairs of fields. This happens because the residual bias acts as an offset in all cases (when a fixed cut-off is chosen) instead of affecting differently each case (as obtained if the maximum amount of data is used in each case). The bias decays with the number of word types used because the more frequent types are responsible for almost all the dissimilarity, specially for *α*=2 [28]. Using 10 000 types as a cut-off, we estimated the textual dissimilarity relative standard deviation, computed over multiple samples of the same scientific field, to be $\hat{\sigma }(D_{\mathrm{lang}})/D_{\mathrm{lang}}\approx 1\%$. Our choice for a larger cut-off of 20 000 types is a conservative choice to ensure a smaller uncertainty of the estimations, i.e. $\hat{\sigma }(D_{\mathrm{lang}})/D_{\mathrm{lang}}<1\%$.

### 5.4. Generalized Jensen–Shannon divergence

Given two texts (indexed by *p* and *q*), we define the probability distributions over all word types *w*=1,…,*V* as **p**=(*p*_*w*_) and **q**=(*q*_*w*_). An information-theoretic measure to quantify their similarity is the generalized Jensen–Shannon divergence:

5.1 $$D_{\alpha }(\text{p},\text{q})=H_{\alpha }\;\left(\frac{\text{p}+\text{q}}{2}\right)-\frac{1}{2}H_{\alpha }(\text{p})-\frac{1}{2}H_{\alpha }(\text{q}),$$

based on the generalized entropy of order *α* (∈R), where

5.2 $$H_{\alpha }(\text{p})=\frac{1}{1-\alpha }\left(\sum_{w}p_{w}^{\alpha }-1\right).$$

Here, we consider a normalized similarity [27]

5.3 $${\tilde{D}}_{\alpha }(\text{p},\text{q})=\frac{D_{\alpha }(\text{p},\text{q})}{D_{\alpha }^{\max}(\text{p},\text{q})}$$

such that ${\tilde{D}}_{\alpha }\in [0,1],$ where $D_{\alpha }^{\max}(\text{p},\text{q})=((2^{1-\alpha }-1)/2)(H_{\alpha }(\text{p})+H_{\alpha }(\text{q})+2/(1-\alpha ))$ is the maximum possible *D*_*α*_ between **p** and **q**, assuming that the set of symbols in each distribution (i.e. the support of **p** and **q**) are disjoint. In other words, if the two corpora have no common word, ${\tilde{D}}_{\alpha }=1$.

Note that, for *α*=1, equation (5.2) yields the Shannon entropy [57], i.e. $H_{\alpha =1}(\text{p})=-\sum_{w}p_{w}\log p_{w}$, and *D*_*α*=1_ is the well-known Jensen–Shannon divergence [58]. Gerlach *et al*. [27] shows that *α*=2 provides the most robust statistical measure of similarity of texts. This motivates our choice of $D_{\mathrm{lang}}={\tilde{D}}_{\alpha =2}$.

## Footnotes

1 Each of the two terms in equation (2.1) can be interpreted as a directed Jaccard distance i→j (j→i) in the sense that we divide the number of edges that are out-links of field *i* (*j*) *and* in-links of field *j* (*i*) by the number of edges that are out-links of field *i* (*j*) *or* in-links of field *j* (*i*).

2 Obtained from 10^3^ bootstrapping samples of each joint distribution *P*(*D*_exp_,*D*_cite_) and *P*(*D*_exp_,*D*_lang_), i.e. comparison of 10^6^ pairs of correlation values.

3 We work at the level of disciplines because most specialities fail to have enough publications in a single year.

## Data accessibility

Deposited at Zenodo https://dx.doi.org/10.5281/zenodo.816303 [55].
